# Neuroinflammatory mechanisms and pharmacological advances in autism spectrum disorder: from inflammatory pathways to targeted interventions

**DOI:** 10.3389/fimmu.2026.1829127

**Published:** 2026-05-20

**Authors:** Xinyu Zhang, Huiqin Xue, Chaoyang Zhu, Chen Ji, Yin Li, Wei Liu

**Affiliations:** 1 Children’s Hospital, Tianjin University, Tianjin, China; 2 Medical School, Tianjin University, Tianjin, China; 3 Department of Research and Education, Tianjin Children’s Hospital, Tianjin, China; 4 Rare Diseases Medical Center, Tianjin Children’s Hospital, Tianjin, China; 5 Tianjin Key Laboratory of Birth Defects for Prevention and Treatment, Tianjin, China

**Keywords:** autism spectrum disorder, blood-brain barrier, maternal immune activation, neuroinflammation, peripheral-to-central immune communication, targeted intervention

## Abstract

**Background:**

Autism spectrum disorder (ASD) is highly diverse in causes and symptoms, and reliable biomarkers and mechanism-based treatment targets are still lacking. Neuroinflammation has been linked to ASD risk, symptom development, and long-term course, but the key “body-to-brain” connections and practical intervention points are not yet clearly organized.

**Methods:**

We synthesize evidence from clinical immune and cerebrospinal fluid studies, neuroimaging, animal models, and multi-omics research, following a mechanistic path from maternal immune activation (MIA) during pregnancy to postnatal immune imbalance, transmission of inflammatory signals to the brain, and subsequent amplification within the central nervous system. We propose the Peripheral-to-Central Inflammatory Cascade-Amplification Model (PC-ICAM) to summarize actionable nodes and recent pharmacological advances.

**Results:**

Within PC-ICAM, ASD-related neuroinflammation follows a cascade from peripheral immune perturbation to peripheral-to-central transmission and central amplification. Peripheral signals may affect the CNS through BBB vulnerability, extracellular vesicle/miRNA communication, and neuro-immune regulatory pathways. Inflammatory signaling involving NF-κB, JAK/STAT, MAPK/ERK, NLRP3, and PI3K-AKT-mTOR may converge with microglial and astrocytic activation, oxidative stress, and mitochondrial dysfunction, disturbing synaptic homeostasis and excitation-inhibition balance. Candidate interventions target IL-6/IL-17 signaling, inflammatory pathways, inflammasome activity, glial modulation, antioxidant/mitochondrial support, BBB stabilization, microbiota-related immune modulation, and exosome-miRNA pathways.

**Conclusions:**

PC-ICAM frames ASD-related neuroinflammation as a traceable and actionable peripheral-to-central cascade with amplification, providing a structured map of mechanisms and therapeutic opportunities. It suggests that precision treatment should not rely on a single anti-inflammatory strategy, but rather combine multi-node, mechanism-matched interventions along the cascade.

## 1 Introduction

Autism spectrum disorder (ASD) is a neurodevelopmental condition with onset in early childhood, characterized by deficits in social communication and the presence of restricted, repetitive behaviors and interests (1). ASD frequently co-occurs with attention-deficit/hyperactivity disorder, anxiety, sleep disturbances, and gastrointestinal dysfunction, reflecting multisystem involvement (2). In recent years, global prevalence has continued to rise (3); for example, in 2022, the U.S. Autism and Developmental Disabilities Monitoring (ADDM) Network reported that ASD prevalence among 8-year-old children was approximately 1 in 31 across 16 surveillance sites in 2022 (4) Beyond its increasing prevalence, ASD typically emerges early in life and persists across the lifespan, imposing substantial and sustained burdens on affected individuals, families, and health and social care systems (2). However, the etiology and pathogenesis of ASD remain incompletely understood. Current diagnosis relies primarily on clinical behavioral assessments, and objective biological markers are lacking (5). This gap not only constrains early identification but also limits the development of effective therapeutic strategies. Accordingly, elucidating disease mechanisms has become an urgent public health and clinical priority; mechanistic advances are essential both for clarifying pathobiology and for enabling the development of targeted pharmacologic and precision intervention approaches.

With the expansion of neuroimmunology research, neuroinflammation has been increasingly recognized as a key pathological component of ASD and a potential entry point for pathway-based drug development (6, 7). Neuroinflammation refers to immune responses within the central nervous system (CNS) elicited by infection, injury, toxic insults, or immune dysregulation, and is characterized by activation of microglia and astrocytes, production of pro-inflammatory mediators such as IL-1β, IL-6, and TNF-α, and disruption of neuroimmune homeostasis (8). Under physiological conditions, controlled inflammatory responses contribute to tissue repair and homeostasis; however, persistent or amplified neuroinflammation can drive neuronal dysfunction, aberrant synaptic remodeling, and network-level injury, with lasting consequences for neurodevelopment (8, 9). Peripheral blood and cerebrospinal fluid studies in ASD have reported altered levels of multiple inflammatory mediators (10, 11). Similarly, postmortem brain studies have provided evidence of microglial and astroglial activation and neuroinflammatory changes in ASD (6, 12). Neuroimaging studies have reported altered TSPO expression in several brain regions in young adult males with ASD (13). Experimental evidence from maternal immune activation models shows that prenatal immune challenge can induce neuroinflammatory changes in the developing brain and contribute to ASD-like behavioral abnormalities in offspring (14). Collectively, these convergent lines of evidence suggest a mechanistic link between neuroinflammation and ASD, whereby glial activation, inflammatory mediator dysregulation, and inflammation-associated disturbances in brain development may impair synaptic organization and neural circuit function, ultimately contributing to atypical neurodevelopment. Nonetheless, the dynamic features of neuroinflammation across developmental stages, immune compartments, and brain regions remain incompletely defined, and the molecular regulatory networks linking inflammation to neural dysfunction require further clarification. Moreover, although anti-inflammatory and immunomodulatory strategies have been explored, a systematic synthesis of existing evidence within a unified mechanistic framework is still lacking.

Against this background, this review summarizes key neuroinflammatory mechanisms implicated in ASD and synthesizes recent advances in mechanism-informed pharmacologic research, then examines how candidate interventions may modulate inflammatory signaling, restore immune homeostasis, and protect neural function, while also considering their current translational relevance to ASD. In summary, we aim to refine the mechanistic framework linking neuroinflammation to ASD and to provide a structured basis for future mechanism-based therapeutic investigation.

## 2 Mechanistic basis of neuroinflammation in ASD

**Figure 1:**
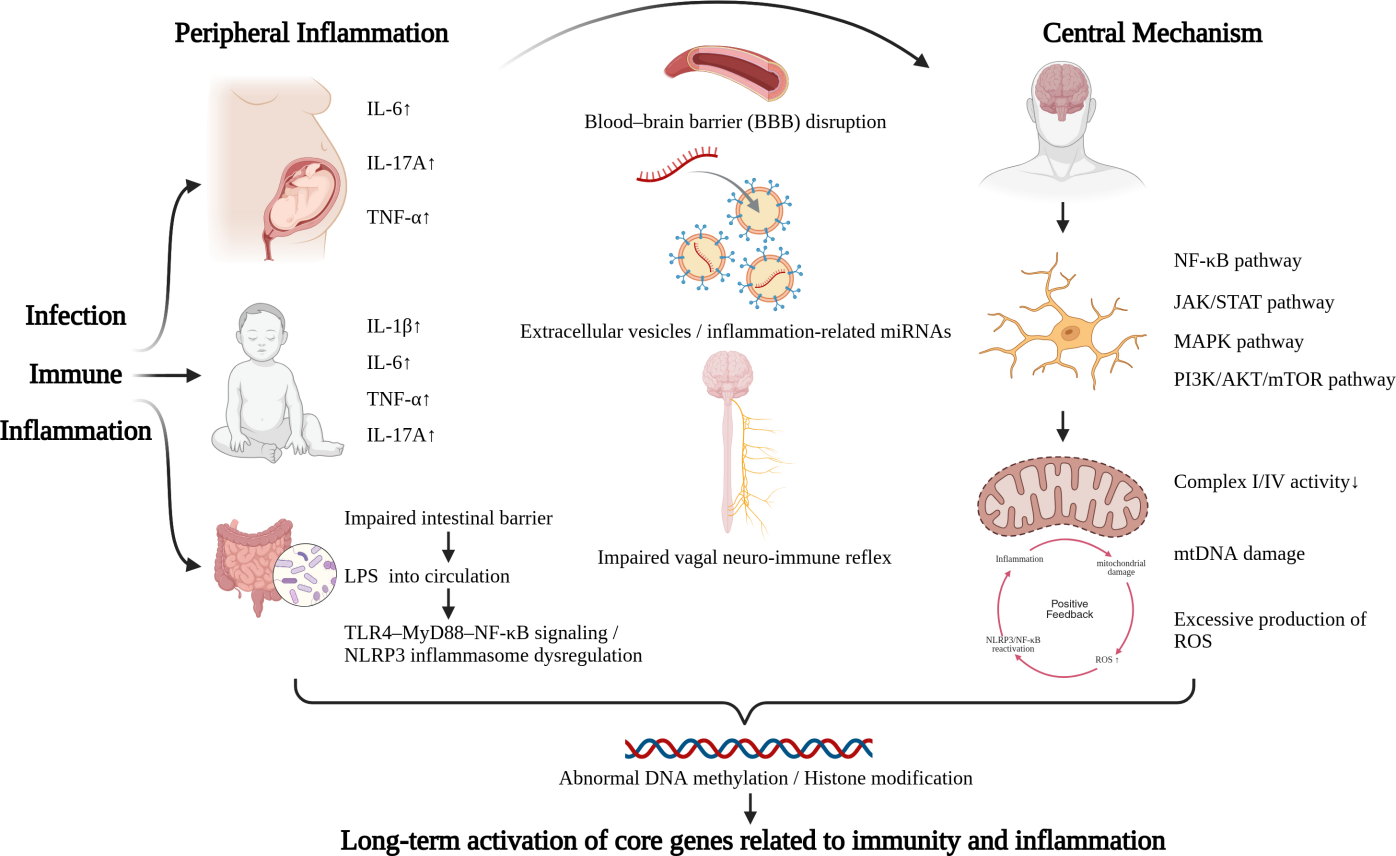
Peripheral-to-central inflammatory cascade–amplification model (PC-ICAM). Peripheral immune activation may arise from prenatal maternal immune activation, postnatal immune dysregulation, infection, inflammatory states, and gut barrier-microbiota disturbances. These processes are associated with increased pro-inflammatory cytokines, including IL-6, IL-17A, TNF-α, and IL-1β, and may promote intestinal barrier dysfunction, LPS translocation, TLR4-MyD88-NF-κB signaling, and NLRP3 inflammasome-related dysregulation. Peripheral inflammatory signals may influence the CNS through BBB vulnerability, extracellular vesicle/miRNA-mediated communication, and neuro-immune regulatory pathways. Within the CNS, coordinated inflammatory signaling involving NF-κB, JAK/STAT, MAPK/ERK, NLRP3, and PI3K-AKT-mTOR may converge with microglial and astrocytic activation, mitochondrial dysfunction, mtDNA damage, and excessive ROS production, forming a self-amplifying inflammatory-oxidative loop. Sustained inflammatory and oxidative stress may further induce epigenetic and transcriptional remodeling, supporting persistent activation of immune-, inflammatory-, and neurodevelopment-related gene programs. (Created with biorender.com).

Neuroinflammation may help explain how peripheral immune disturbances are translated into abnormal brain development and function in ASD. Accumulating evidence suggests that this process is not driven by a single event, but rather unfolds across multiple developmental stages and biological levels. During pregnancy, maternal immune activation (MIA) can affect the fetal brain through placental immune signaling, whereas after birth, peripheral immune dysregulation and chronic low-grade inflammation may continue to sustain and amplify inflammatory activity within the CNS. In parallel, glial activation, persistent inflammatory signaling, oxidative stress, mitochondrial dysfunction, synaptic abnormalities, and blood-brain barrier disruption interact to shape a self-reinforcing pathogenic process. On this basis, we propose a Peripheral to Central Inflammatory Cascade-Amplification Model (PC-ICAM) to integrate these interconnected mechanisms. The proposed PC-ICAM framework is illustrated in **Figure 1**.This model provides a structured framework for understanding how peripheral immune perturbations may initiate, propagate, and amplify neuroinflammatory responses in ASD, and it serves as the basis for the six mechanistic dimensions discussed in the following sections. **Table 1** summarizes the key mechanistic evidence supporting each component of the PC-ICAM framework.

**Table 1:** Mechanistic evidence supporting the PC-ICAM framework in ASD-related neuroinflammation.

Section	Core concept	Role in PC-ICAM	Representative references
2.1 Immune activation	Maternal immune activation and postnatal peripheral immune dysregulation may establish a sustained pro-inflammatory susceptibility state in ASD.	Upstream initiation layer that primes immune–neural vulnerability and provides the peripheral inflammatory input for subsequent cascade amplification.	Atladóttir et al., 2010 (15); Zerbo et al., 2015 (16); Choi et al., 2016 (17); Shin Yim et al., 2017 (18); Hsiao et al., 2013 (19); Ashwood et al., 2011 (10).
2.2 Inflammatory signaling and immune-regulatory imbalance	Peripheral and central immune abnormalities may converge on NF-κB, JAK/STAT, MAPK/ERK, NLRP3 and PI3K/AKT/mTOR signaling, together with Th17/Treg disequilibrium.	Molecular amplification layer that converts immune activation into persistent inflammatory signaling and weakens immune-regulatory control.	Naik et al., 2011 (20); Shmarina et al., 2020 (21); Hughes et al., 2022 (22, 23); Ahmad et al., 2018 (24); Cheng et al., 2017 (25); Onore et al., 2017 (26).
2.3 Peripheral-to-central immune signal propagation	Peripheral inflammatory signals may influence the CNS through BBB dysfunction, extracellular vesicle/miRNA delivery and impaired vagal neuro–immune reflexes.	Transmission layer that links peripheral immune imbalance to central neuroinflammatory responses.	Rasile et al., 2022 (27); Versele et al., 2022 (28); Shigemoto-Mogami et al., 2018 (29); Qin et al., 2022 (30); Zhang et al., 2025 (31); Gök Dağıdır et al., 2025 (32).
2.4 Glial activation and neuroimmune imbalance	Microglia and astrocytes may shift from homeostatic states toward reactive phenotypes, releasing inflammatory mediators and disturbing synaptic and metabolic support.	Central amplification layer that translates incoming inflammatory signals into neuron–glia dysfunction, synaptic remodeling abnormalities and circuit-level vulnerability.	Morgan et al., 2010 (33); Suzuki et al., 2013 (34); Falcone et al., 2021 (35); Vakilzadeh et al., 2022 (36); Russo et al., 2018 (37); Mansur et al., 2021 (38).
2.5 Mitochondrial dysfunction and oxidative stress	Sustained inflammation and glial activation may promote mitochondrial dysfunction, ROS/RNS accumulation, electron transport chain impairment and mtDNA injury.	Metabolic reinforcement layer in which oxidative stress and mitochondrial injury feedback to maintain inflammasome and cytokine signaling.	Chauhan et al., 2011 (39); Gu et al., 2013 (40); Tang et al., 2013 (41); Rose et al., 2012 (42); Giulivi et al., 2010 (43); Kato et al., 2023 (44); Maier et al., 2023 (45).
2.6 Epigenetic regulation and immune memory-like persistence	Chronic inflammatory and oxidative stress may induce epigenetic and post-transcriptional reprogramming, including trained immunity–like persistence.	Persistence layer that explains how transient or fluctuating inflammatory exposures may leave long-term immune and glial vulnerability.	Shulha et al., 2012 (46); Bakulski et al., 2021 (47); García-Ortiz et al., 2021 (48); Saeliw et al., 2022 (49); Perini et al., 2023 (50); Nguyen et al., 2025 (51).

### 2.1 Immune activation

From a developmental perspective, immune abnormalities in ASD can be conceptualized as a continuous immune activation axis extending from fetal-stage MIA to persistent postnatal peripheral immune hyperreactivity. These two phases are temporally distinct but mechanistically connected, together functioning as an upstream driver of ASD-related neuroinflammation within the PC-ICAM.

MIA refers to an abnormal maternal immune response during pregnancy—triggered by infection, inflammation, or immune imbalance—and is characterized by increased production of pro-inflammatory cytokines that may affect fetal neurodevelopment through placental immune signaling and cytokine-mediated fetal brain effects (52–54). Epidemiological evidence links maternal infection, chronic immune-mediated inflammatory conditions such as inflammatory bowel disease, and autoimmune disease during or around pregnancy to increased ASD risk in offspring (15, 16, 55, 56).

Consistent with these observations, many studies have shown that maternal exposure to viral or bacterial mimetics such as poly(I:C) or LPS induces robust increases in circulating inflammatory cytokines, including IL-6, IL-17A, and TNF-α (17, 18, 57, 58). The IL-17A-IL-17RA pathway has emerged as a key mediator: IL-17A derived from maternal Th17 cells can act on the fetal cortex, disrupt local neurodevelopment, and induce social behavioral abnormalities, whereas blockade of IL-17A signaling substantially ameliorates these phenotypes (17, 58), supporting the view that systemic immune activation is an early and biologically plausible upstream event in ASD-related developmental vulnerability. Maternal gut microbiota composition may further shape the fetal immune milieu by modulating maternal immune homeostasis and cytokine profiles (59, 60), whereas microbiota-targeted interventions can partially improve offspring inflammatory and behavioral abnormalities (19, 61).

Beyond MIA, postnatal peripheral immune abnormalities are also important drivers of neuroinflammation in ASD. Postnatal peripheral immune dysregulation in ASD may be sustained by repeated infectious exposures and persistent gut barrier-microbiota disturbances (62–64). This state is typically characterized by sustained immune activation, dysregulated cytokine profiles, and immune cell functional disturbances, manifesting as enhanced pro-inflammatory responses, impaired immune regulation, and maintenance of chronic low-grade inflammation (10, 65–67).

Multiple studies have reported a recurrent pro-inflammatory peripheral immune phenotype in children with ASD, including altered serum/plasma cytokine profiles with increased IL-1β, IL-6, TNF-α-related signals, and elevated IL-17A, suggestive of Th17-related immune skewing (10, 65, 67–69). In addition, evidence of Th/Treg-related transcriptional dysregulation, Th17/Treg imbalance, and reduced TGF-β1-related immunoregulatory signaling suggests a shift away from immune homeostasis in ASD (27, 28, 70, 71). The monocyte lineage also shows abnormal responsiveness to TLR ligands, together with altered monocyte subpopulations and activation-marker expression, supporting a pro-inflammatory myeloid bias in ASD (66, 72, 73).

Dysregulation of the gut-brain-immune axis may further sustain peripheral immune imbalance. Clinical studies in ASD have reported increased intestinal permeability-related markers, including zonulin, I-FABP, LPS, and LBP, suggesting impaired gut barrier integrity and enhanced exposure to gut-derived microbial products (74–76). Experimental evidence further indicates that gut barrier disruption can increase circulating LPS and activate TLR4-MyD88-NF-κB signaling, while other studies also support peripheral TLR4-associated immune/oxidative abnormalities and NLRP3/inflammasome-related inflammatory dysregulation (77–81). Together, these findings suggest a “dysbiosis-barrier dysfunction-immune activation-neuroinflammation” loop that may sustain peripheral inflammatory signaling and facilitate its propagation toward the CNS.

Together, these findings indicate that both prenatal immune perturbations induced by MIA and chronic postnatal peripheral immune activation can create a long-standing pro-inflammatory susceptibility state that primes abnormal CNS responses, driving downstream molecular and cellular pathology.

### 2.2 Activation of inflammatory signaling and breakdown of immune regulation

MIA and postnatal peripheral immune abnormalities may converge in ASD to establish a sustained pro-inflammatory state accompanied by coordinated activation of multiple inflammatory signaling pathways. Rather than functioning as isolated abnormalities, these pathways are more likely to operate as an interconnected inflammatory network that amplifies peripheral immune responses, weakens immune homeostasis, and facilitates peripheral-to-central inflammatory transmission.

Among these pathways, NF-κB-linked innate immune activation appears to constitute an important upstream inflammatory hub in ASD. Original studies have shown increased NF-κB DNA-binding activity in peripheral blood cells from children with autism (20), while additional evidence also supports heightened NF-κB-related inflammatory transcription in peripheral immune cells (21). In parallel, monocytes from children with ASD exhibit exaggerated responses to TLR ligands, transcriptomic analyzes of activated monocytes further support dysregulation of inflammatory-response programs, and more recent studies have identified altered activated monocyte populations and monocyte subpopulation shifts associated with immune activation in ASD (22, 23, 65, 70, 82). Taken together, these findings are consistent with a hyper-responsive myeloid phenotype in which NF-κB-related inflammatory transcription may help sustain peripheral inflammatory tone and prime downstream cytokine-driven signaling cascades in ASD (20–23, 65, 70, 82).

Cytokine-driven signaling pathways provide major amplification arms for this inflammatory state. In MIA models, maternal IL-17A signaling induces abnormal cortical development and autism-like behaviors in offspring, supporting a causal role for this pathway in linking prenatal immune perturbation to abnormal neurodevelopment (56, 83). In BTBR autistic mice, attenuation of JAK1-STAT3 activation reduces pro-inflammatory cytokine expression, whereas in MIA-induced autism-like mice, inhibition of the miR-301a/SOCS3/STAT3 axis reduces inflammatory signaling and ameliorates autism-like behavioral abnormalities (24, 84).

MAPK signaling, particularly ERK-related cascades, appears to represent another convergent pathway through which inflammatory or cellular stress may be linked to neurobehavioral abnormalities in ASD. Evidence from ASD-relevant experimental models has shown aberrant MAPK/ERK activation, including elevated ERK signaling during early neurodevelopment, while peripheral lymphocytic studies in individuals with ASD also support dysregulation of this pathway (25, 85–87).

A further layer of inflammatory amplification in ASD may be provided by inflammasome activation. Original studies have reported activation of multiple inflammasome complexes together with increased IL-1β- and IL-18-related inflammatory output, while more recent work in peripheral immune cells and ASD-related cellular systems further links NLRP3 activation to mitochondrial dysfunction and oxidative stress (80, 81, 88). Emerging mechanistic evidence also supports a functional contribution of microglial NLRP3 signaling to repetitive behavioral abnormalities, suggesting that inflammasome activation may connect inflammatory activation with ROS accumulation, metabolic injury, and persistent neuroinflammatory stress in ASD (89).

Inflammatory signaling may also converge on metabolic and growth-related pathways in ASD. Experimental studies in ASD-relevant models suggest that IL-6 can enhance PI3K-AKT/mTOR-related signaling in hippocampal neurons, whereas human studies support dysregulation of the Akt/mTOR pathway in individuals with ASD (26, 90–92).

As these inflammatory cascades persist, immune-regulatory balance may weaken at the same time. Original studies in children with ASD support a shift away from immune homeostasis and toward pro-inflammatory disequilibrium, reflected by abnormalities in Th17/Treg balance, reduced Foxp3-associated regulatory activity, and lower TGF-β1-related immunoregulatory signaling (70, 71, 93, 94).

In summary, inflammation in ASD is unlikely to reflect a single-pathway abnormality. This pro-inflammatory state is repeatedly transmitted and reinforced between the periphery and CNS, establishing an inflammatory foundation for glial activation, metabolic stress, and neurocircuit injury.

### 2.3 Peripheral-to-central immune signal propagation

Peripheral inflammatory signals can influence the CNS via multiple routes, establishing immune communication between peripheral and central compartments in ASD. A key mechanism involves increased BBB permeability: developmental inflammatory exposure can impair brain vascular integrity, while pro-inflammatory cytokines, particularly IL-1β and TNF-α, can alter brain endothelial junctional organization and increase BBB permeability. These changes may compromise BBB integrity and facilitate peripheral inflammatory signaling into the brain parenchyma, where activated microglia can further disrupt the BBB and amplify cytokine/chemokine production (27–29).

Beyond barrier disruption, extracellular vesicles and circulating miRNAs may serve as refined molecular carriers linking peripheral immune activation to CNS responses. Peripheral inflammatory exosomes and small extracellular vesicles can contribute to neuroinflammation, cross the BBB, and induce glial activation (95, 96). In ASD, serum extracellular vesicles are increased and can stimulate human microglia to secrete IL-1β, while L1CAM-captured vesicles show altered transcriptomic and proteomic profiles, and dysregulated exosomal miRNA profiles have also been reported (30, 31, 97, 98). These findings suggest that vesicle-mediated signaling may reshape recipient-cell inflammatory and transcriptional programs and contribute to peripheral-to-central immune propagation.

In ASD-related animal models, transcutaneous auricular vagus nerve stimulation can modulate inflammatory responses and improve ASD-like behavioral abnormalities, suggesting that neuro-immune reflex pathways may influence inflammatory control in ASD (32, 99).

This cross-system amplification may help explain the link between peripheral inflammation and CNS immune abnormalities and provides a rationale for interventions targeting peripheral-to-central inflammatory signaling.

### 2.4 Glial activation and neuroimmune imbalance

Under sustained inflammatory drive, CNS glial cells transition from homeostatic states toward pro-inflammatory and/or reactive phenotypes, and this shift is considered a major accelerator of persistent neuroinflammation and neurocircuit injury in ASD (33–36).

In pro-inflammatory states, activated microglia may release cytokines and oxidative mediators that disturb neuronal homeostasis, synaptic regulation, and circuit maturation. In ASD, postmortem and neuroimaging studies provide convergent evidence of microglial abnormalities, including developmental microglial priming in the temporal cortex, increased microglial activation and density in the dorsolateral prefrontal cortex, and increased microglial activation in multiple brain regions detected by PET imaging (12, 33, 34).

Astrocytic abnormalities have also been reported in ASD, including layer-specific changes in glial cell number and reduced astrocyte number with increased activation state in the prefrontal cortex (35, 36). Beyond histopathological evidence, patient-derived iPSC studies further suggest that ASD astrocytes may disrupt neuronal development, synaptogenesis, and spontaneous neuronal activity, accompanied by increased inflammatory gene expression, including IL6 (37).

Additional evidence indicates complement dysregulation and reduced expression of astrocytic glutamate transporter genes, including SLC1A2 and SLC1A3, suggesting impaired glutamate clearance (38, 100). Moreover, ASD-derived astrocytes can destabilize neuronal network activity through aberrant Ca²^+^ signaling, supporting a mechanistic link between astrocyte dysfunction, synaptic disruption, and excitatory-inhibitory imbalance in ASD (100, 101).

Together, these findings suggest that altered microglial and astrocytic states may interact to amplify neuroimmune imbalance and disrupt neuron-glia communication in ASD, although the detailed microglia-astrocyte feedback mechanisms and their causal contribution to circuit-level dysfunction remain to be fully clarified (12, 33–38, 100, 101).

### 2.5 Mitochondrial dysfunction and oxidative stress

As inflammation persists and glial reactivity intensifies, mitochondria—central to energy metabolism and redox homeostasis—become vulnerable to inflammation-associated injury and may actively contribute to the maintenance of chronic neuroinflammation.

In postmortem brain tissue from individuals with ASD, region-specific reductions in mitochondrial electron transport chain activity, altered pyruvate dehydrogenase activity, increased mitochondrial DNA (mtDNA) copy number, and mtDNA deletions have been reported in the frontal and temporal cortex, indicating impaired oxidative phosphorylation and abnormal mitochondrial compensation (39–41). Oxidative damage and inflammation associated with low glutathione redox status have also been observed in the autism brain (42).

Studies in peripheral cells from children with ASD have shown increased mitochondrial ROS production, oxidative damage to mtDNA, reduced reserve capacity, and impaired bioenergetic responses, further supporting systemic redox-related mitochondrial vulnerability in ASD (43, 102). These abnormalities are also supported by *in vivo* imaging evidence showing lower mitochondrial complex I availability in the anterior cingulate cortex in ASD and by magnetic resonance spectroscopy findings of elevated cerebral lactate in autistic adults, consistent with altered brain energy metabolism (44, 45). Under sustained reciprocal amplification, a self-perpetuating loop may emerge in which mitochondrial dysfunction and ROS production activate inflammatory signaling, which in turn further aggravates mitochondrial injury (80).

In summary, mitochondrial dysfunction and oxidative stress constitute a key metabolic node in neuroinflammatory progression. They not only transmit upstream effects of inflammatory pathways and glial activation, but also sustain pro-inflammatory output through mitochondria-associated oxidative and inflammatory signaling, forming a self-reinforcing “inflammation-oxidative stress” cycle that may drive neurocircuit injury and behavioral phenotype development in ASD.

### 2.6 Epigenetic regulation and immune memory

Under long-term exposure to inflammation and oxidative stress, innate immune cells and CNS glia can undergo transcriptional and epigenetic reprogramming. Even when external stimuli diminish or resolve, cells may remain hyper-responsive to pro-inflammatory cues, facilitating the conversion of transient inflammation into persistent neuroinflammation.

Mechanistically, inflammatory signaling may alter DNA methylation, chromatin accessibility, and transcriptional activation of immune- and neurodevelopment-related genes. In ASD brain tissue, activation-associated epigenetic marks—such as altered enrichment or peak morphology of H3K4me3 at neuro- and immune-related loci-have been observed, suggesting tissue- and developmental stage-specific epigenetic remodeling (46). Recent epigenomic studies extend this view to regulatory chromatin architecture. ATAC-seq mapping of the developing human brain has identified temporally and regionally specific open chromatin elements, including regulatory regions linked to ASD-risk genes (47). ChIP-seq analyzes of ASD-associated transcriptional regulators further showed convergent binding at open-chromatin and H3K27ac-marked loci, suggesting shared regulatory disruption across ASD-related gene networks (48). Although BisChIP-seq has not yet been widely applied in ASD cohorts, related methylation–chromatin profiling approaches may help jointly assess DNA methylation, histone modification, and chromatin accessibility in ASD-relevant contexts (49).

Prospective birth-cohort evidence further shows that DNA methylation profiles at birth in maternal blood, cord blood, and placenta are associated with subsequent ASD risk and enriched for ASD-related genes (50). Peripheral blood studies also report altered global and locus-specific DNA methylation, including LINE-1, NCAM1, and NGF methylation patterns (103), while LINE-1 and Alu methylation signatures have been associated with the expression of ASD-related genes (104). In ASD-discordant sibling pairs, integrated methylation and gene-expression analysis further identified methylation changes linked to neurogenesis, synaptic organization, and immune-cell composition (51).

Oxidative and inflammatory stress may reinforce epigenetic and post-transcriptional dysregulation in ASD. In peripheral blood neutrophils from children with ASD, reduced DNMT1 expression and DNA hypomethylation were associated with increased inflammatory mediators, including CCR2 and MCP-1 (105). Meanwhile, ASD has been associated with dysregulated miRNA profiles in biofluids; salivary profiling identified multiple differentially expressed miRNAs in children with ASD, with predicted target networks involving developmental and signaling pathways (106). Transcriptional and epigenetic analyzes in human microglia indicate that MEF2C-regulated networks overlap with ASD risk-related programs, suggesting that microglial epigenetic dysregulation may contribute to the long-term maintenance of neuroimmune abnormalities (107).

Overall, these changes may reshape immune- and neurodevelopment-related gene programs, providing a regulatory substrate through which transient inflammatory insults could evolve into persistent neuroimmune vulnerability in ASD.

## 3 Pharmacological advances targeting neuroinflammatory mechanisms in ASD

Within the PC-ICAM framework, neuroinflammation in ASD can be viewed as a progressive process linking prenatal immune perturbation, postnatal peripheral immune imbalance, and central glial amplification. On this basis, pharmacologic strategies are increasingly being developed to target specific nodes along this cascade, rather than relying solely on symptomatic management. The following sections summarize recent advances in mechanism-based interventions across four major dimensions of this inflammatory network. **Table 2** summarizes candidate pharmacological strategies according to the PC-ICAM-related mechanisms they are proposed to target.

**Table 2:** Mechanism-guided pharmacological strategies targeting ASD-related neuroinflammation.

Section	Intervention/strategy	Core concept	Existing application context	Representative references
3.1 Prenatal immunomodulatory interventions	Maternal cytokine-axis modulation (IL-17A and IL-6/IL-6R)	Maternal cytokine signaling provides a biologically plausible link between MIA and altered fetal neurodevelopment.	IL-17A blockade or related modulation has been tested mainly in MIA mouse models; IL-6R blockade has clinical exposure data from selected pregnant patients with critical COVID-19 or inflammatory diseases, and newer IL-6R modulation has been tested in prenatal inflammation models.	Choi et al., 2016 (17); Andruszewski et al., 2025 (83); Isaac et al., 2023 (105); Nana et al., 2024 (106).
3.1 Prenatal immunomodulatory interventions	Indication-based anti-inflammatory treatment during pregnancy	Glucocorticoids can suppress excessive maternal inflammatory activation, but fetal neurodevelopmental risks require careful consideration.	Clinically used in defined obstetric or maternal inflammatory contexts, including COVID-19-positive pregnancy care, but not as neurodevelopmental preventive therapy.	Elgormus et al., 2024 (108); Laugesen et al., 2025 (109); Gyllenhammer et al., 2021 (110).
3.1 Prenatal immunomodulatory interventions	Prenatal redox and nutritional modulation	N-acetylcysteine, vitamin E, vitamin D, and ω-3-related strategies may influence oxidative stress, placental inflammation, and neurodevelopmental vulnerability.	Supported by placental redox studies, vitamin D supplementation in MIA-related pregnancy models, and cohort evidence on maternal fish or ω-3 intake; most use contexts are nutritional or adjunctive rather than ASD-specific.	Lofthouse et al., 2021 (111); Jhamb et al., 2023 (112); Wang et al., 2025 (113); Lyall et al., 2024 (114).
3.1 Prenatal immunomodulatory interventions	Maternal microbiota-based interventions	Probiotics and prebiotics may alter maternal immune tone, milk immune composition, infant microbiome development, and offspring neurodevelopmental trajectories.	Explored in maternal probiotic/prebiotic supplementation, maternal dysbiosis models, breastmilk immune-composition studies, infant microbiome studies, and premature-infant neurodevelopment contexts.	Siegler Lathrop et al., 2024 (115); Hudobenko et al., 2025 (116); Gonia et al., 2024 (117); Baucells et al., 2023 (118); Divakara et al., 2024 (119).
3.2 Targeting pro-inflammatory pathways and peripheral immunity	Sulforaphane/Nrf2-related redox-inflammatory modulation	Sulforaphane may regulate redox balance and inflammatory responses, with clinical signals for behavioral improvement in ASD.	Tested directly in ASD clinical trials as a redox- and inflammation-modulating intervention, making it one of the more clinically proximal candidates.	Singh et al., 2014 (120); Zimmerman et al., 2021 (121).
3.2 Targeting pro-inflammatory pathways and peripheral immunity	Cytokine-signaling pathway modulation (JAK/STAT and MAPK/p38 MAPK)	JAK/STAT and MAPK signaling connect cytokine-driven inflammation with cellular stress and neurodevelopmental phenotypes.	Investigated mainly in ASD-related animal models, including STAT3 inhibition in BTBR mice and p38α MAPK inhibition in the SERT Ala56 mouse model.	Ahmad et al., 2019 (122); Robson et al., 2018 (123).
3.2 Targeting pro-inflammatory pathways and peripheral immunity	P2X7–NLRP3–IL-1β axis modulation	P2X7 inhibition may reduce inflammasome-related IL-1β signaling and microglial inflammatory activation.	Supported by maternal P2X7 inhibition in ASD-related mouse offspring and by broader neuroimmune models of P2X7/NLRP3 regulation, including autoimmune neuritis and microglial-cell studies.	Szabó et al., 2022 (124); Xie et al., 2024 (125); Campagno and Mitchell, 2021 (126).
3.2 Targeting pro-inflammatory pathways and peripheral immunity	PI3K/AKT/mTOR pathway modulation	mTOR-related signaling links immune activation, autophagy, synaptic pruning, and neurodevelopmental phenotypes.	Has translational precedent in tuberous sclerosis complex and Tsc2-related models, including everolimus evaluation for TSC-associated neuropsychiatric manifestations.	Ehninger et al., 2008 (127); Tang et al., 2014 (128); Krueger et al., 2017 (129).
3.3 Interventions targeting peripheral-to-central immune transmission	Barrier-directed strategies, including minocycline-related BBB protection	Barrier-protective approaches may limit the entry or amplification of peripheral inflammatory signals in the CNS.	Evidence comes mainly from non-ASD neuroinjury models, including traumatic brain injury and subarachnoid hemorrhage, where minocycline-related AQP4 or microvascular protection has been examined.	Lu et al., 2022 (130); Gendosz de Carrillo et al., 2023 (131).
3.3 Interventions targeting peripheral-to-central immune transmission	S1P/S1P1-related modulation	S1P/S1P1 signaling can regulate BBB permeability and inflammatory immune-cell trafficking.	Clinically grounded by multiple-sclerosis-related S1P modulation and experimentally examined in BBB disruption after traumatic brain injury, epilepsy, and intracerebral hemorrhage.	Nishihara et al., 2015 (132); Zhang et al., 2022 (133); Yang et al., 2023 (134); Feng et al., 2024 (135).
3.3 Interventions targeting peripheral-to-central immune transmission	Exosome/miRNA-directed modulation	Inflammation-related vesicle and miRNA signaling may represent a modifiable carrier system for peripheral-to-central immune communication.	Currently exploratory; ASD evidence includes extracellular vesicle/exosomal biomarker studies and an ASD-related rat model in which plasma exosomal miR-30b-5p attenuated neuroinflammation.	Zheng et al., 2025 (136).
3.4 Targeting glial activation and neuroimmune imbalance	Microglia-focused anti-inflammatory modulation (minocycline)	Minocycline may suppress excessive microglial inflammatory activity and modulate microglial polarization.	Supported by an ASD mouse model and by broader neurological or inflammatory settings, including obesity-related hypothalamic microglial activation, subarachnoid hemorrhage, cerebral ischemia-reperfusion injury, brain trauma, and chronic pain.	Luo et al., 2023 (137); Coker et al., 2022 (138); Wang et al., 2024 (139); Mohammadian et al., 2025 (140).
3.4 Targeting glial activation and neuroimmune imbalance	PPAR-γ agonism (pioglitazone)	Pioglitazone may exert glial and immune-metabolic modulatory effects through PPAR-γ activation.	Clinically used as a metabolic drug and tested as adjunctive treatment with risperidone in children with ASD; animal evidence suggests possible IL-6-related mechanisms.	Kirsten et al., 2018 (141); Ghaleiha et al., 2015 (142).
3.4 Targeting glial activation and neuroimmune imbalance	Astrocyte/glutamate-homeostasis modulation (ceftriaxone and EAAT2–GLT-1 enhancement)	Astrocyte-targeted glutamate clearance may reduce excitotoxic stress and support neuroimmune homeostasis.	Derived mainly from antibiotic repurposing and glutamate-transporter studies, including neuroprotection through increased glutamate transporter expression and EAAT2/GLT-1 modulation in astrocytes or young-rat models.	Zaitsev et al., 2019 (143); Rothstein et al., 2005 (144); Lee et al., 2008 (145).

### 3.1 Prenatal immunomodulatory interventions

MIA is regarded as an important environmental contributor to early ASD risk, making prenatal immune modulation a potential preventive strategy. Experimental studies indicate that interference with the maternal IL-17A pathway can attenuate MIA-related cortical and behavioral abnormalities in offspring, supporting the biological intervenability of this axis (17, 83). By contrast, evidence for IL-6-targeted intervention remains indirect and is derived mainly from other pregnancy-associated inflammatory conditions rather than ASD-specific prevention studies. Available observational data have not identified strong maternal-fetal safety signals with anti-IL-6R antibody exposure during pregnancy (108, 109), while newer IL-6R modulators have shown anti-inflammatory and fetal-protective effects in prenatal inflammation models (110). However, no randomized clinical trials have tested primary prevention of ASD in humans.

Glucocorticoids can regulate excessive immune activation during pregnancy, but their use should remain strictly indication-based. Observational evidence has linked prenatal systemic glucocorticoid exposure to modest increases in adverse offspring neuropsychiatric outcomes, while maternal inflammatory responsivity and glucocorticoid sensitivity may vary across gestational stages and populations (111–113). Accordingly, glucocorticoids should be reserved for clear maternal indications and used at the lowest effective dose for the shortest possible duration under specialist supervision.

Beyond cytokine- or pathway-directed immunomodulation, several adjunctive strategies have been explored to reduce prenatal inflammatory burden. Antioxidant approaches such as N-acetylcysteine and vitamin E are mechanistically relevant to placental redox regulation, but direct evidence that they prevent adverse neurodevelopmental outcomes remains limited (114, 115). Among nutritional interventions, prenatal vitamin D supplementation has shown anti-inflammatory and behavioral benefits in MIA-related models, while higher maternal intake of ω-3-rich fish has been associated with lower risk of autism-related outcomes in offspring (116, 117). Microbiota-based approaches, including probiotics and prebiotics, may help modulate the maternal gut ecosystem, milk immune composition, and infant microbial development. Animal and early maternal-infant studies suggest potential downstream effects on offspring immune and neurodevelopmental trajectories, although the evidence remains preliminary (118–121, 137).

In summary, immune modulation centered on the IL-6/IL-17A axis, judicious glucocorticoid use, and antioxidant, nutritional, and microbiota interventions all show signals consistent with potential intervenability.

### 3.2 Targeting pro-inflammatory pathways and peripheral immunity

Among broad redox- and inflammation-related interventions, sulforaphane has shown behavioral benefits in clinical studies of ASD, whereas minocycline improves autism-related behaviors by modulating microglial polarization in an ASD mouse model (122–124).

Importantly, although organism-level human prevention studies are lacking, human cellular models provide complementary mechanistic evidence for pharmacological intervenability. For example, sulforaphane-related studies in human peripheral blood mononuclear cells showed increased cytoprotective and heat-shock response markers while reducing pro-inflammatory markers such as IL-6, IL-1β, COX-2, and TNF-α, including in samples from individuals with ASD (127). In addition, ASD-related human iPSC-derived neuronal models have shown that targeted pathway modulation can partially rescue disease-relevant cellular phenotypes, including abnormal neuronal development, synaptic deficits, altered morphology, and impaired neuronal network activity (128–130).

JAK/STAT and MAPK pathways have also emerged as plausible targets: STAT3 inhibition reduces immune activation and inflammatory cytokine signaling in the BTBR mouse model, while p38α MAPK inhibition reverses brain and gastrointestinal phenotypes in the SERT Ala56 mouse (131, 132). In parallel, inflammasome-related mechanisms have attracted attention, as maternal P2X7 receptor inhibition prevents autism-like phenotypes in male offspring through the NLRP3-IL-1β pathway (133).

The PI3K/AKT/mTOR pathway represents another important node linking immune signaling with synaptic and metabolic dysfunction, supported by evidence from Tsc2+/- models, mTOR-dependent autophagy-related synaptic pruning deficits, and clinical exploration of mTOR inhibition in tuberous sclerosis complex-associated neuropsychiatric manifestations (134–136).

### 3.3 Interventions targeting peripheral-to-central immune transmission

Pharmacological strategies targeting peripheral-to-central immune transmission remain largely at the stage of mechanistic exploration. Current efforts focus mainly on two processes: preservation of BBB integrity and modulation of exosome/miRNA-mediated inflammatory signaling. Minocycline is of particular interest because, beyond its anti-inflammatory effects, non-ASD neuroinjury studies suggest barrier- or microvascular-protective properties, including modulation of AQP4-related injury responses and preservation of brain microvascular ultrastructure (138, 146). Similarly, S1P/S1P1-related signaling has emerged as another candidate BBB-regulatory pathway: fingolimod and selective S1P1 modulators can attenuate BBB disruption in experimental settings, while additional studies indicate that the Sphk1/S1P/S1P1 axis participates in BBB permeability regulation and barrier breakdown under pathological conditions (139–141, 147). Although these approaches have not yet been established in ASD, they support the concept of barrier-directed intervention as an upstream strategy for limiting neuroinflammatory amplification.

Exosome- and miRNA-based approaches remain at an early exploratory stage. In an ASD-related rat model, plasma exosomal miR-30b-5p was reported to attenuate neuroinflammation, supporting the potential intervenability of vesicle-mediated inflammatory signaling (142). However, ASD-specific molecular signatures, delivery strategies, and pharmacodynamic biomarkers remain insufficiently defined, and clinical translation is still preliminary.

### 3.4 Targeting glial activation and neuroimmune imbalance

Interventions aimed at suppressing excessive glial activation and restoring neuroimmune homeostasis have become an increasingly important therapeutic direction.

Among currently explored agents, minocycline remains one of the most studied glia-related candidates. In an ASD mouse model, it improved autism-related behaviors by modulating microglial polarization; however, evidence from non-ASD neurological and clinical settings suggests that its effects on neuroinflammation and neural injury may vary with disease stage and pathological context (125, 126, 143–145). Pioglitazone, a PPAR-γ agonist, represents another candidate glial or immune-metabolic modulator: preclinical studies show improvement of autism-like behaviors, and adjunctive clinical use with risperidone has shown benefits in irritability-related domains (148, 149).

Astrocyte-related strategies have mainly focused on glutamate homeostasis. Ceftriaxone can enhance EAAT2/GLT-1-related glutamate clearance, supporting a mechanistic rationale for targeting astrocyte-mediated excitotoxicity, although ASD-specific therapeutic evidence remains limited (150–152). In addition, emerging targets such as the P2X7 receptor may allow more selective regulation of microglial inflammatory activity and functional state, although current evidence remains largely preclinical and mostly non-ASD-specific (153, 154).

Collectively, glia-targeted pharmacology is transitioning from non-specific anti-inflammatory approaches toward more refined, cell type- and pathway-informed modulation; however, translation will depend on consistent inflammatory stratification, biomarker readouts, and reproducible clinical endpoints.

## 4 Discussion

The COVID-19 pandemic provides a recent epidemiological context for considering infection-related immune activation and offspring neurodevelopmental risk. U.S. ADDM surveillance showed that developmental evaluations and ASD identification were transiently disrupted during the early pandemic, with later recovery, suggesting that observed changes may partly reflect health-care access, referral patterns, and diagnostic ascertainment rather than true incidence shifts (4). The COMBO cohort found no increase in M-CHAT-R autism screening positivity among pandemic-born or prenatally exposed children, and the ASPIRE cohort found no association with abnormal neurodevelopmental screening through 24 months (155, 156). By contrast, retrospective electronic health record studies reported possible risk signals for early neurodevelopmental diagnoses, while a large Northern California cohort identified a sex-specific association with ASD risk among female offspring (157–159). Thus, current evidence does not establish a definitive post-COVID increase in ASD incidence. COVID-19 should be viewed as an important natural epidemiological context requiring longer ASD surveillance.

In summary, the PC-ICAM model is developed from an ASD-centered pathological perspective and provides a mechanistic framework to explain how peripheral immune dysregulation contributes to neurodevelopmental alterations. Specifically, it describes a cascade process in which peripheral immune perturbations are initiated, transmitted to the central nervous system, and subsequently amplified through barrier dysfunction, glial activation, metabolic alterations, and epigenetic regulation. While this framework is grounded in ASD, it may also offer a useful conceptual approach for understanding other neurological disorders involving immune dysregulation.

Existing ASD models have emphasized genetic susceptibility, synaptic dysfunction, altered cortical connectivity, maternal immune activation(MIA), gut-brain axis disruption, and gene-environment interactions (52, 160–166). Rather than replacing these perspectives, PC-ICAM connects them by describing how peripheral immune perturbations may be initiated, transmitted to the CNS, and amplified through barrier, glial, metabolic, and epigenetic mechanisms.

Within this integrative context, PC-ICAM also shows conceptual overlap with the two-hit hypothesis which proposes that an early vulnerability, such as genetic susceptibility or prenatal immune exposure, may interact with later environmental or inflammatory challenges to increase neurodevelopmental risk (52, 160, 161). Recent double-hit models support this concept by combining ASD-relevant genetic variation with MIA, or MIA with postnatal immune stimulation, although outcomes vary according to developmental timing, sex, genetic background, and the nature of the second challenge (160, 161). The above hypothesis remains an important framework in ASD research for conceptualizing such interactions. However, PC-ICAM differs in that it further specifies the biological processes through which immune-related perturbations are propagated and amplified across peripheral and central systems in ASD.

PC-ICAM also shows conceptual overlap with gut-brain axis frameworks, although its emphasis is distinct. Gut-brain axis models emphasize bidirectional communication among the microbiome, intestinal barrier, microbial metabolites, immune signaling, neuroendocrine pathways, and the CNS. Multi-level analyzes have identified ASD-associated microbial, metabolic, cytokine, dietary, and brain gene-expression profiles, while also highlighting limited reproducibility and strong cohort heterogeneity (165). PC-ICAM incorporates gut-brain disruption as one potential upstream or sustaining source of peripheral immune activation, particularly in individuals with gastrointestinal comorbidity, altered microbial metabolites, barrier dysfunction, or chronic low-grade inflammation.

Importantly, PC-ICAM should not be interpreted as a universal model for all individuals with ASD. ASD is highly heterogeneous at clinical, genetic, developmental, and molecular levels. Recent phenotypic and genetic analyzes have identified clinically relevant ASD classes linked to distinct molecular programs, supporting subtype-oriented mechanistic models (166). Accordingly, PC-ICAM may be most applicable to biologically defined subgroups characterized by maternal inflammatory exposure, recurrent postnatal immune activation, gastrointestinal comorbidity, abnormal cytokine profiles, oxidative or mitochondrial stress, BBB-related vulnerability, or glial inflammatory signatures.

PC-ICAM is best understood as a complementary framework that links immune, barrier, glial, metabolic, and epigenetic mechanisms into a peripheral-to-central amplification sequence. Its value lies not in explaining all ASD cases, but in clarifying how inflammatory risk may be initiated and sustained in specific ASD subgroups and how this process may intersect with genetic, synaptic, gut-brain, and two-hit models.

Immune-targeted interventions should be interpreted not only according to their mechanistic targets, but also according to the contexts in which they have already been used. Many candidate strategies discussed in this review were not originally developed for ASD, and their relevance differs substantially depending on whether the evidence comes from ASD clinical studies, ASD-related animal models, genetically defined neurodevelopmental syndromes, pregnancy-associated inflammatory conditions, or broader immune-mediated and neurological diseases.

This distinction is important because mechanistic plausibility does not necessarily indicate clinical readiness. Interventions supported by ASD clinical evidence may be considered closer to symptom- or subgroup-oriented repurposing, whereas findings from ASD-related animal models mainly provide mechanistic proof-of-concept. In contrast, evidence extrapolated from maternal inflammatory diseases, autoimmune conditions, neuroinjury, epilepsy, multiple sclerosis, or other non-ASD contexts should be viewed as context-informing rather than ASD-specific. Such evidence may help define feasibility, safety boundaries, therapeutic timing, and potential biomarkers, but it cannot directly establish efficacy for ASD prevention or treatment.

Therefore, the therapeutic implications of PC-ICAM should be understood as a translational hierarchy rather than a direct therapeutic recommendation. Future studies should integrate inflammatory stratification, developmental timing, target-engagement biomarkers, and long-term neurodevelopmental outcomes to determine which patients, at which stage, and under which inflammatory conditions may benefit from immune-modulatory interventions.

Besides that, high-throughput complementary medicine screening may broaden the source of ASD intervention candidates by enabling systematic identification of natural compounds with multi-target anti-inflammatory and neuroimmune-modulatory potential. Rather than replacing pharmacologic treatment, natural-product libraries could be screened using PC-ICAM related readouts, including microglial cytokine release, NF-κB/Nrf2 balance, NLRP3 inflammasome activation, mitochondrial redox status, BBB integrity, and glia-mediated synaptic pruning (167–169). Sulforaphane provides a translational example of a natural compound with antioxidant and immunometabolic effects that has entered ASD clinical testing, although efficacy remains variable (122, 123). Therefore, complementary screening should be considered a hypothesis-generating platform that requires compound standardization, mechanistic validation, toxicity testing, pharmacokinetic assessment, and controlled clinical trials before application.

## 5 Conclusion

The evidence reviewed here supports neuroinflammation as an important mechanistic dimension of ASD, particularly in biologically susceptible subgroups, rather than as a universal explanation for all cases. Within the PC-ICAM framework, ASD-related neuroinflammation can be conceptualized as a peripheral-to-central cascade in which prenatal immune perturbations, postnatal peripheral immune dysregulation, gut barrier-microbiota disturbances, and chronic low-grade inflammation create an upstream pro-inflammatory susceptibility state. These peripheral signals may then influence the CNS through BBB vulnerability, extracellular vesicle/miRNA-mediated communication, and neuro-immune regulatory pathways, thereby establishing a biological bridge between systemic immune imbalance and central neuroimmune activation.

The pharmacological evidence summarized in this review further suggests that neuroinflammation-related mechanisms are potentially intervenable, although clinical translation remains at an early stage. Candidate strategies include prenatal modulation of IL-6/IL-17-related immune pathways, antioxidant and nutritional approaches, microbiota-directed interventions, inhibition of pro-inflammatory signaling pathways, inflammasome modulation, redox and mitochondrial support, BBB-directed protection, exosome/miRNA-related approaches, and glia-focused regulation. However, much of the current evidence is derived from animal models, mechanistic studies, non-ASD neurological contexts, or small clinical trials, and therefore does not yet establish broad therapeutic efficacy for ASD.

Accordingly, PC-ICAM should be viewed as a complementary framework that connects immune, barrier, glial, metabolic, epigenetic, genetic, synaptic, gut-brain, and two-hit perspectives rather than replacing existing ASD models. Its main value lies in clarifying how inflammatory risk may be initiated, propagated, and amplified in specific ASD subgroups, and in identifying actionable nodes for mechanism-guided intervention. Future research should prioritize biomarker-defined inflammatory subtyping, longitudinal validation of peripheral-to-central immune pathways, multimodal CNS readouts, and rigorously designed mechanism-matched clinical trials. Such efforts will be essential for determining whether neuroinflammation-targeted strategies can move beyond biological plausibility toward precise and clinically meaningful interventions for ASD.
